# Supplementary material for: ERK1/2 is an ancestral organising signal in spiral cleavage
Source: Nat Commun. 2022 Apr 28;13:2286. doi: 10.1038/s41467-022-30004-4 (PMC9050690; doi:10.1038/s41467-022-30004-4)
Supplement: Supplementary file 1 — Supplementary Information [file 41467_2022_30004_MOESM1_ESM.pdf]

Supplementary Information

ERK1/2 is an ancestral organising signal in spiral cleavage

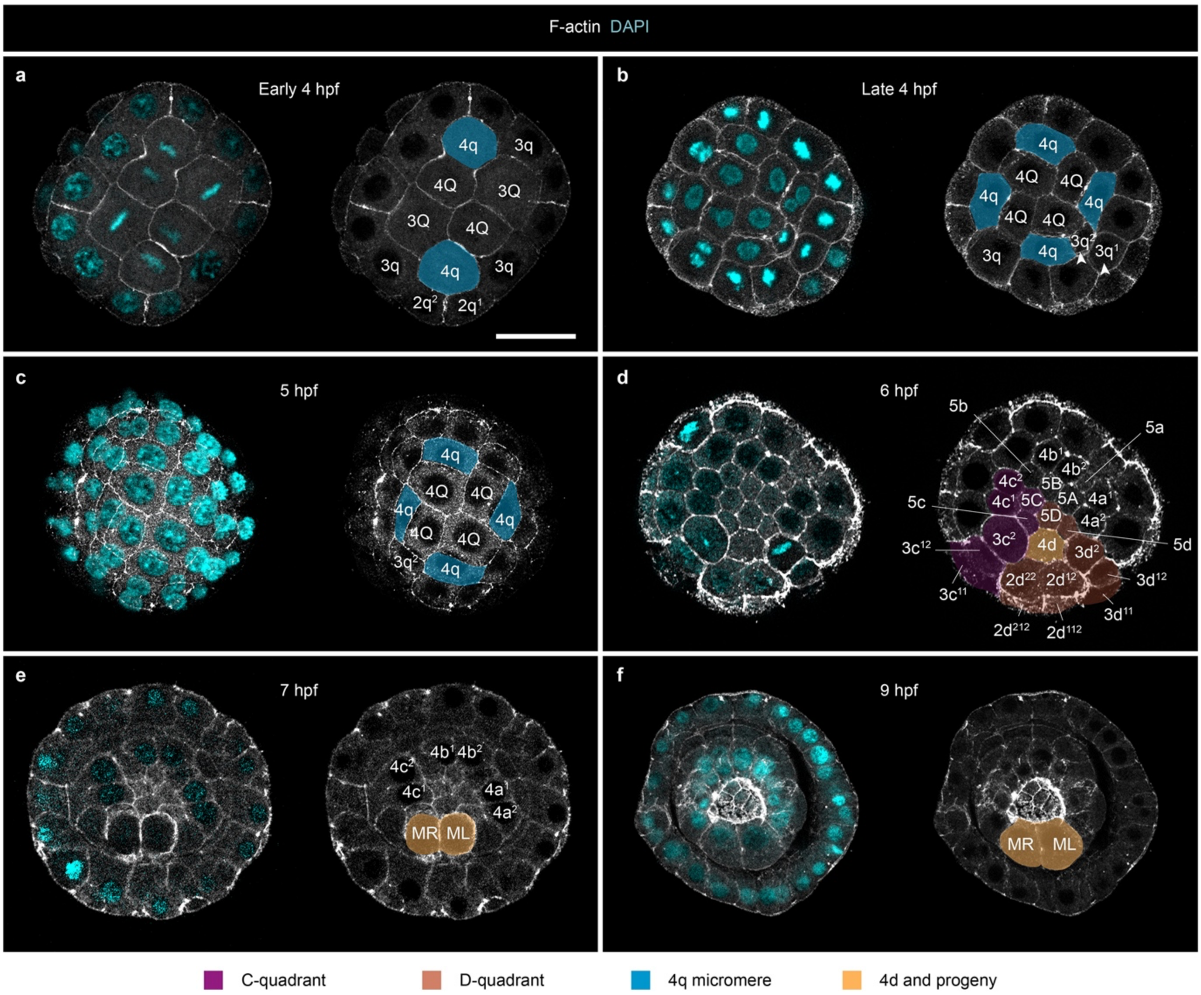

Supplementary Figure 1. Dynamics of cell division of the fourth micromere quartet in *O. fusiformis*. a–f z-stack confocal projections of the vegetal pole of whole mount embryos stained with phalloidin (F-actin, gray; it shows cell boundaries) and DAPI (nuclei) during the onset and early division of the fourth micromere quartets and the blastomere inferred to be the 4d cell based on ERK1/2 activity. The fourth micromere quartet emerges asymmetrically (early 4 hours post fertilisation, hpf), which is soon followed by the asymmetric division of one of the 3q micromeres (arrowheads) (late 4 hpf). The vegetal pole is however quatri-radial at 5 hpf, with the 4d arresting its division with respect to 4a–c, and only cleaving into MR

and ML (4d daughter cells) after ingression at 7 hpf. False colouring indicates cell or quadrant identities. Descriptions for **a–f** are based on at least 10 embryos per stage, from a minimum of two biological replicates. Scale bar is 50 µm.

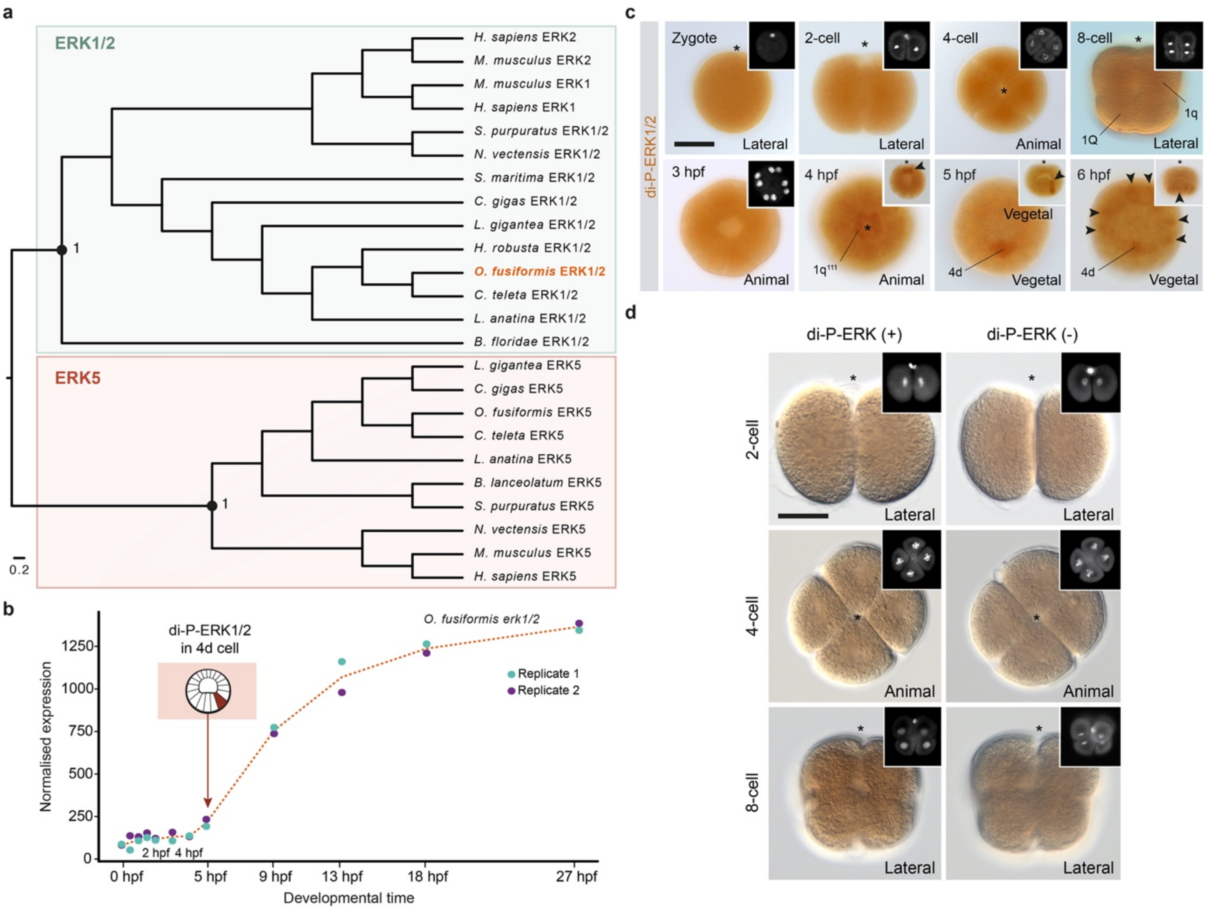

Supplementary Figure 2. ERK1/2 activity during *O. fusiformis* early embryogenesis. a Maximum likelihood orthology assignment for *O. fusiformis* ERK1/2*,* using ERK5 as outgroup. Only bootstrap values for major nodes are shown. **b** Normalised expression of *erk1/2* during *O. fusiformis* development, from the active oocyte to the mitraria larval stage in hours post fertilisation (hpf). The time of the specification of 4d is highlighted with a schematic drawing. Coloured dots indicate values of expression for each replicate. The dotted red line shows the mean value of expression. *erk1/2* expression starts to increase at 4 hpf, which is consistent with the enrichment of di-phosphorylated-ERK1/2 in specific blastomeres from 4 hpf onwards (see Figure 2). **c** Whole mount immunohistochemistry against di-phosphorylated-ERK1/2 (di-P-ERK1/2; dark orange) during spiral cleavage (from the zygote to 6 hpf) in *O. fusiformis*. Low background levels are detected during early divisions (zygote to 3 hpf). Later, di-P-ERK1/2 is enriched in the four $1q^{111}$ animal micromeres at 4 hpf, the 4d

micromere at 5 hpf and in six vegetal cells forming a bilaterally symmetrical pattern (arrowheads) plus 4d at 6 hpf. Insets show either nuclear staining (gray; zygote to 3 hpf) or lateral views (4 to 6 hpf). **d** Whole mount immunohistochemistry against di-P-ERK1/2 and the corresponding negative control (without primary antibody). Background levels are comparable between the two conditions. Insets show nuclear staining (gray). In **c** and **d**, asterisks point to the animal pole. Descriptions for **c** and **d** are based on at least 10 embryos per stage, from a minimum of two biological replicates. Scale bars are 50 μm.

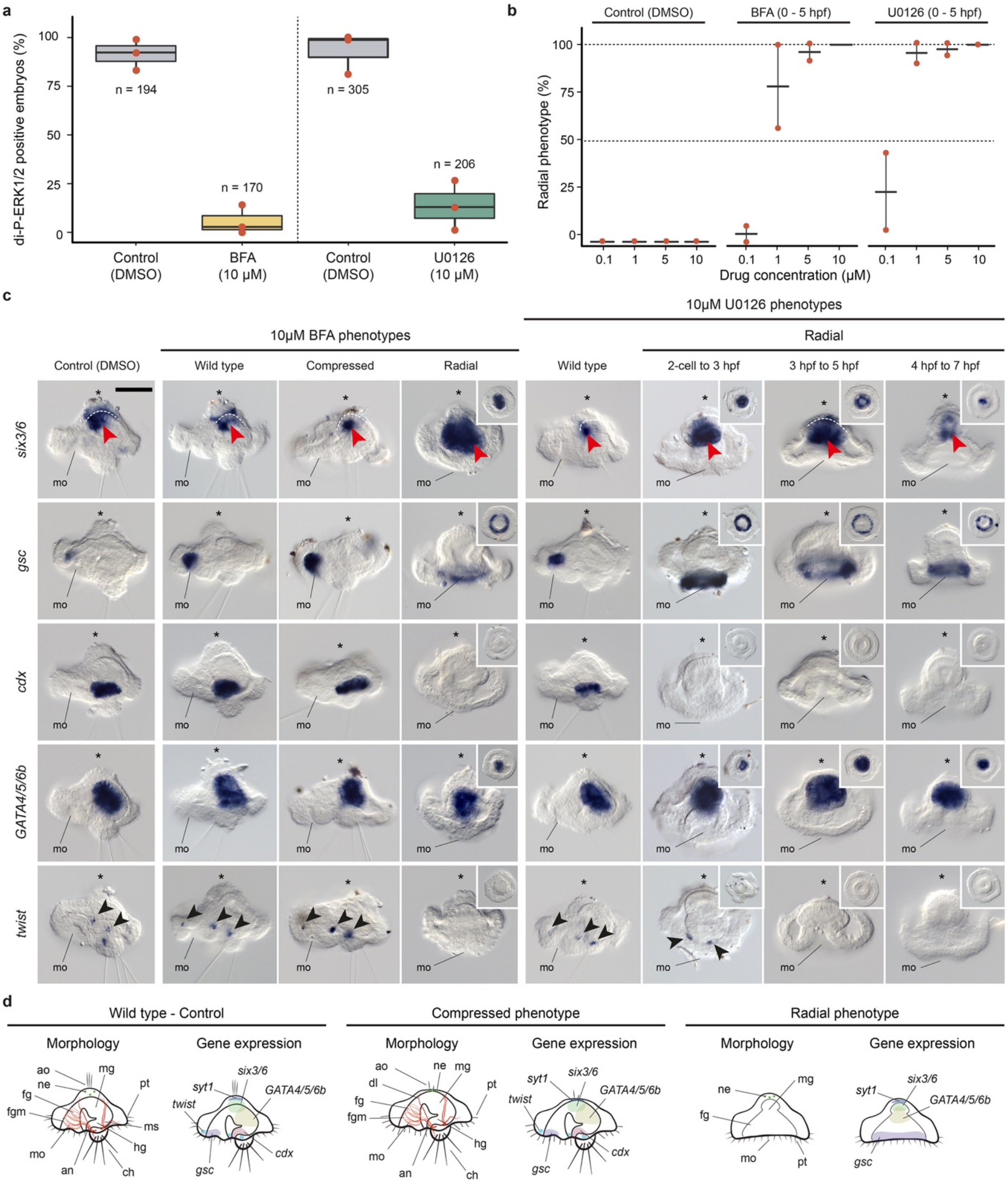

Supplementary Figure 3. The effect of BFA and U0126 treatment in *O. fusiformis* development. a Boxplots depicting the median percentage of coeloblastulae showing di-phosphorylated ERK1/2 (di-P-ERK1/2) enrichment in the 4d micromere after 0.5 to 5 hours post fertilisation (hpf) treatment with brefeldin A (BFA), U0126 or DMSO (control). Red dots indicate the values for each experimental replicate. **b** Plot indicating the percentage of radial phenotypes obtained when treating embryos from 0.5 to 5 hpf with a range of BFA and

U0126 concentrations. A concentration of 10 µM shows the greatest penetrance and was thus used throughout the study (N=2, error bars= sd).**c** Whole mount *in situ* hybridisation of apical (*six3/6*), oral (*gsc*), posterior/hindgut (*cdx*), midgut (*GATA4/5/6b*) and trunk mesodermal (*twist*) gene markers in control and treated embryos fixed at larval stage. Radial phenotypes have a reduction of the apical marker (although *six3/6* endodermal expression is maintained and/or expanded; red arrowheads; the dotted white line separates ectodermal from endodermal expression), radial expansion of an oral gene, loss of posterior structures (chaetae) and posterior/trunk mesoderm gene expression and retain the expression of a midgut gene. Radial larvae treated with U0126 from the 2-cell stage to 3 hpf show scattered *twist* expression in some embryos (black arrowheads) and embryos treated from 4 to 6 hpf are seemingly more elongated along the apical-oral axis. The compressed larval phenotype after BFA treatment from 0.5 to 4 hpf have a reduced apical organ and apical tuft (first row) and reduced expression of apical markers (*six3/6*), but otherwise normal morphology besides an obliterated internal blastocele. Asterisks indicate the apical pole. Insets are ventral views. **d** Schematic drawings (not to scale) of the control and treated larval phenotypes, depicting the morphological landmarks, genes and gene expression patterns considered to ascribe treatment outcomes to each of the phenotypic categories (summarised in Supp. Table 3). For **c**, Supplementary Data 2 reports the detailed numbers. Scale bars are 50 µm. an: anus; ao: apical organ; ch: chaetae; dl: dorsal levator muscles; fg: foregut; fgm: foregut muscles; hg: hindgut; mg: midgut; mo: mouth; ms: muscles; ne: neurons; pt: prototroch.

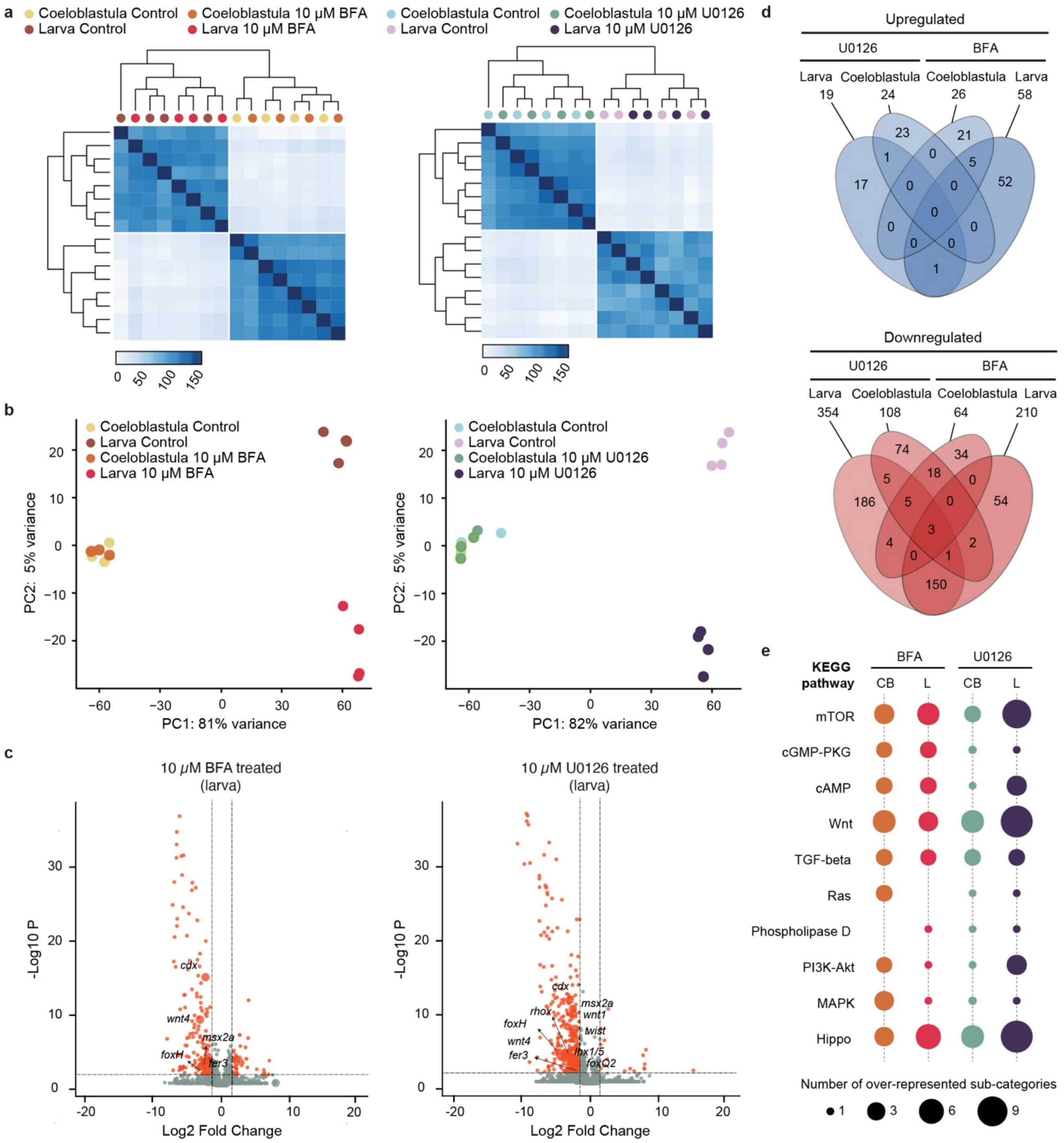

Supplementary Figure 4. Differential expression analyses in BFA and U0126 treated embryos. a Hierarchically clustered pairwise correlation matrix between brefeldin A (BFA) and U0126 treated and control samples. All replicates of the same condition are highly correlated. Scale shows the relative difference in Euclidean distance. **b** Principal component (PC) analysis plot for BFA (left) and U0126 (right) RNA-seq analyses. Coeloblastula and larval samples are clearly separated, as well as treated and control samples fixed at larval stage, which reflects the increase in the number of differentially expressed genes at this later

time point compared to the coeloblastula stages (as shown in panel **d**). **c** Volcano plots for BFA and U0126 treated embryos studied at 24hpf (larval stage). Red dots show differentially expressed (DE) genes and analysed candidate genes are labelled in each comparison. In both conditions, most DE genes are downregulated (two-sided Wald test). **d** Venn diagrams showing the number of up- (top) and downregulated (bottom) DE genes shared between different conditions. **e** Ten most overrepresented KEGG pathways in each of the four conditions analysed by RNA-seq. The size of the coloured dots is proportional to the number of overrepresented sub-categories nested into a given KEGG pathway.

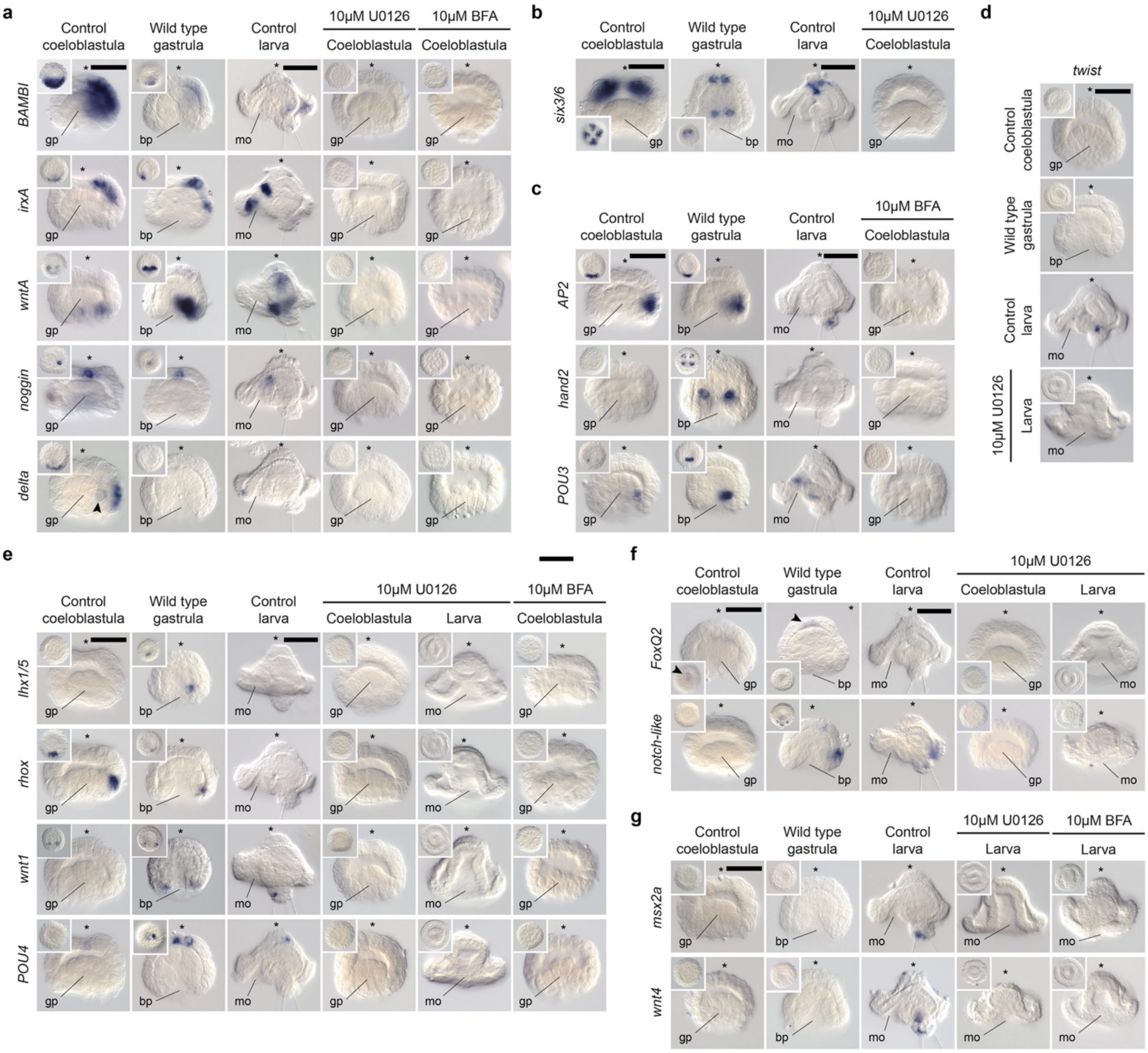

Supplementary Figure 5. Validation of the differential expression analyses. a–**g** Whole mount *in situ* hybridisation of control and treated embryos, as well as wild type gastrula stage to reconstruct the time course of expression of each gene. **a** Candidate genes differentially expressed at the coeloblastula stage in both BFA and U0126 treatments. *BAMBI*, *irxA* and *delta* are expressed in the dorsal ectoderm and 4d (only *delta*) at the coeloblastula stage. *WntA* is expressed in the posterior blastoporal lip and probably larval mesoderm, and *noggin* is detected in the apical ectoderm of the blastula and gastrula. **b** *six3/6* (expressed at the apical ectoderm and larval oesophagus) is differentially expressed at the coeloblastula stage in U0126 treatment only. **c** Candidate genes differentially expressed at the coeloblastula stage in BFA treatment only. *AP2* is expressed in posterodorsal ectoderm, and *hand2* and *POU3*

are likely expressed in mesodermal cell types. **d** *twist* (expressed in mesoderm) is differentially expressed at the larval stage in U0126 treatment only. **e** Candidate genes differentially expressed at the coeloblastula stage in both BFA and U0126 treatments, as well as at the larval stage in U0126 treatment. *Lhx1/5* and *rhox* are expressed in mesodermal cells, *wnt1* is detected in posterior ectodermal cells and *POU4* in apical ectodermal cells. **f** Candidate genes differentially expressed at the coeloblastula and larval stages in U0126 treatment. *foxQ2* is expressed in the apical ectoderm and *notch-like* is detected in the posterodorsal ectoderm of the gastrula and larva. **g** Candidate genes differentially expressed at the larval stage in both BFA and U0126 treatments. *Msx2a* is expressed in the posterior ectoderm and *wnt4* in posterior mesoderm. In all cases, the expression patterns disappear in BFA and U0126 treated embryos compared to the control condition at the relevant stages. Main panels are lateral views and insets are ventral views (except for genes expressed apically, where insets are apical views). Asterisk point to the animal/apical pole. For **a**–**g**, Supplementary Data 2 reports the detailed numbers. Arrowheads point to 4d in **a** and to the apical organ in **f**. Scale bars are 50 μm. bp: blastopore; gp: gastral plate; mo: mouth.

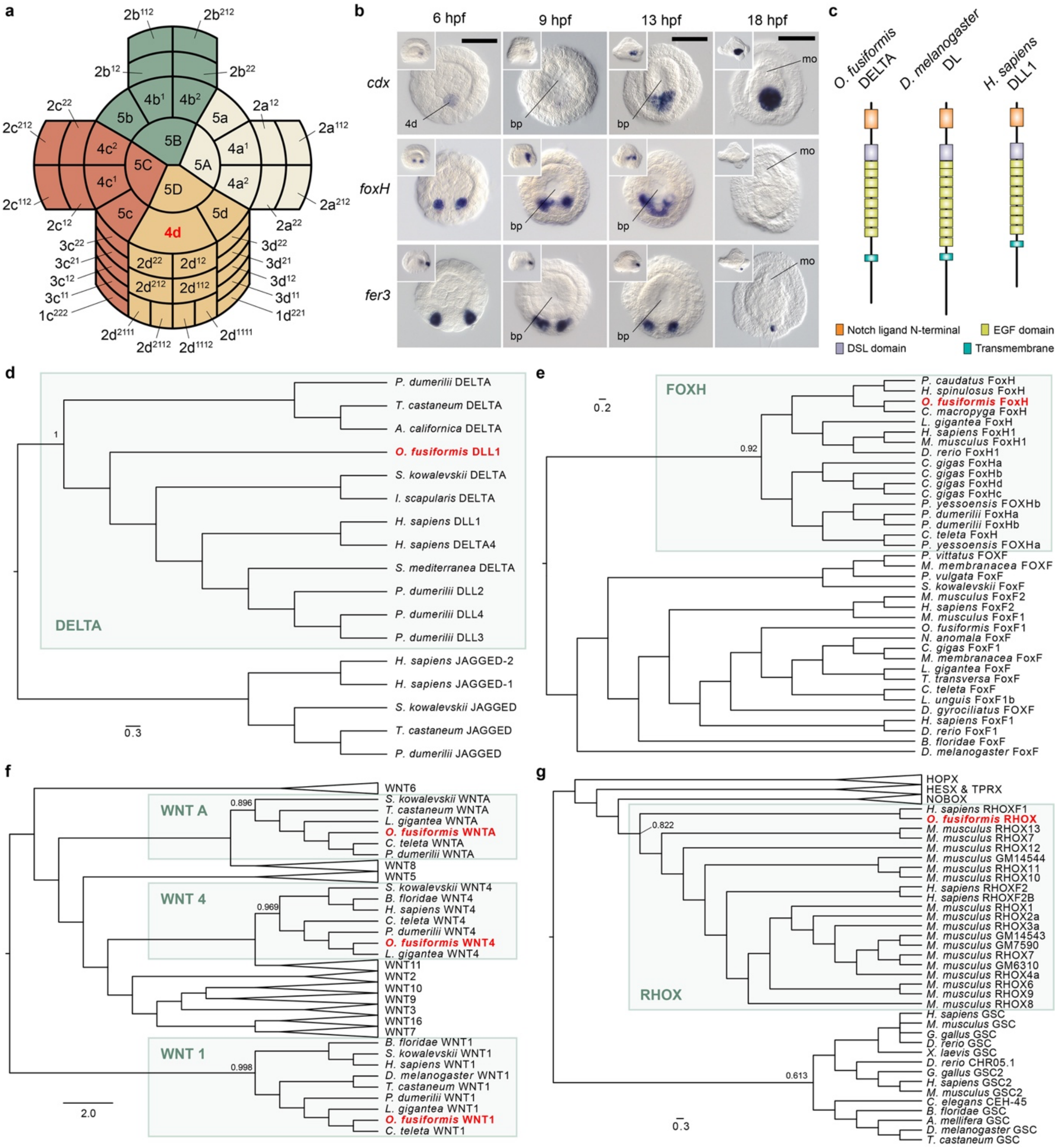

Supplementary Figure 6. Genes patterning the D-quadrant after the specification of the 4d-organiser cell. a Schematic drawing of the cellular arrangements and cell nomenclature of the vegetal pole just after the specification of the 4d micromere in *O. fusiformis* embryo at 5.5 hours post fertilisation (hpf). Each colour depicts a different quadrant. **b** Ventral views of the time course of expression of *cdx*, *foxH* and *fer3* via whole mount *in situ* hybridisation from 6 hpf to 18 hpf (early larval stage) during *O. fusiformis* embryogenesis (insets show lateral views). While *cdx* is expressed in the cells forming the hindgut, *foxH* appears to

upregulate at the earliest stages of cells giving rise to mesodermal derivatives. The gene *fer3* is expressed in two posterior most cells of unknown function in the gastrula and larva. Insets are lateral views. **c** Schematic drawing of the protein domain architecture of the Notch ligand DELTA in *O. fusiformis* in comparisons with the domain structures of the Delta orthologs in *D. melanogaster* and *H. sapiens*. **d–g** Maximum likelihood orthology assignments of DELTA, FOXH, WNT ligands, and RHOX in *O. fusiformis*. Only bootstrap values supporting each major clade are shown. Descriptions for **b** are based on at least 10 embryos per stage, from a minimum of two biological replicates. In **b**, bp stands for blastopore and mo for mouth. Scale bars are 50 μm.

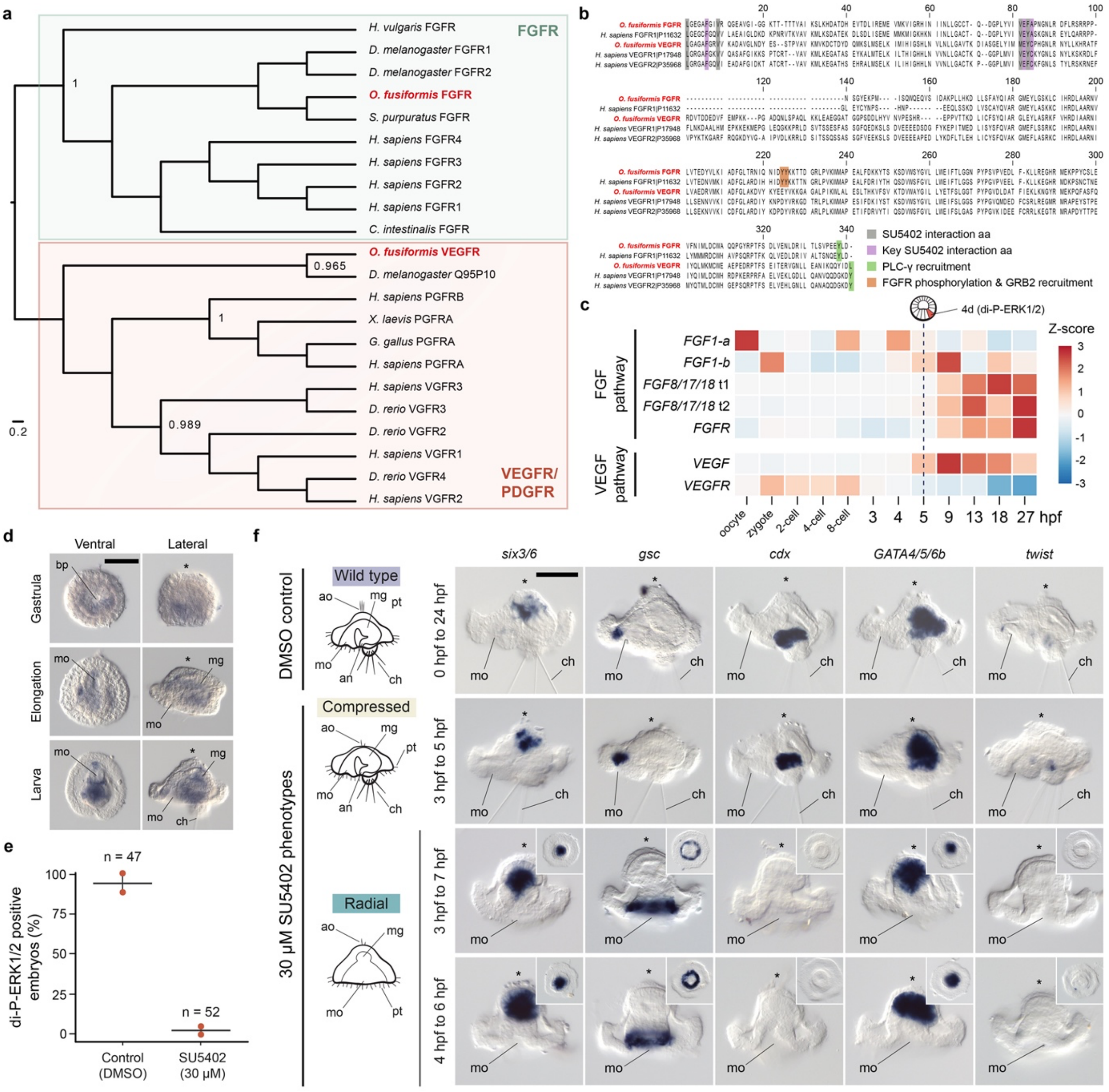

Supplementary Figure 7. The FGF pathway and FGFR activity during 4d specification in *O. fusiformis*. a Maximum likelihood orthology assignment of fibroblast growth factor receptor (FGFR) and vascular endothelial growth factor receptor (VEGFR). Only bootstrap values for key nodes are shown. **b** Multiple protein alignment between *O. fusiformis* and *H. sapiens* FGFR and VEGFR orthologs indicating conserved residues for the interaction of the drug SU5402 with these receptors. **c** Temporal time course of expression of members of the FGF and VEGF pathways in *O. fusiformis*. The heatmap depicts the normalised z-score value of expression for each gene. The vertical dotted line highlights the specification of the 4d micromere. **d** Whole mount *in situ* hybridisation time course of expression of *fgfr* from

gastrula to larval stages in *O. fusiformis*. At the gastrula stage, *fgfr* is expressed in the posterior archenteron wall. During organogenesis and elongation, as well as at the larval stage, *fgfr* is detected in putative mesodermal tissues and midgut. **e** Plot depicting the proportion of treated and control embryos exhibiting di-phosphorylated ERK1/2 enrichment in the 4d cell at 5 hours post fertilisation (hpf) after SU5402 drug treatment from 0.5 to 5 hpf (N=2, error bars= sd). **f** Morphological and molecular characterisation of SU5402 treated embryos fixed at the larval stage. The first column shows diagrams of lateral views of the wild type, compressed and radial phenotypes. From the second column onwards, whole mount *in situ* hybridisations of control and SU5402 treated conditions. The "compressed" larvae are similar to brefeldin A (BFA) "compressed" larvae, with reduced apical organ and apical tuft (second row) and reduced expression of apical markers (*six3/6*), but other tissues are not affected. Radial larvae are phenocopies of BFA and U0126 radial larvae with reduction of apical markers (*six3/6*) in the apical ectoderm, radial expansion of oral genes (*gsc*), loss of posterior structures (chaetae) and expression of posterior endomesodermal markers (*cdx*, *twist*), but normal expression of an endodermal gene (*GATA4/5/6b*). Insets are ventral views. Asterisks point to the animal/apical pole. Descriptions for **d** are based on at least 10 embryos per stage, from a minimum of two biological replicates. For **e**, Supplementary Table 2 reports the detailed numbers. For **f**, Supplementary Table 12 reports the detailed numbers. Scale bars are 50 µm. ao: apical organ; an: anus; bp: blastopore; ch: chaetae; gp: gastral plate; mg: midgut; mo: mouth; pt: prototroch.

Supplementary Table 1. The role of di-P-ERK1/2 in spiralians

Reference	Clade	Species	Type of cleavage	Description of di-P-ERK1/2 immunoreactivity during early development	Axial defects after MAPK inhibition	U0126 concentration	Phenotype
REF[1]	Cheilostomatida (Bryozoa)	*Membranipora membranacea*	Non spiral (biradial)	3D	Yes	1,10 or 25 µM	But, presumably maternal, prior to the activation in 3D.
REF[2]	Gastropoda (Mollusca)	*Haliotis asinina*	Equal	3D	Yes	10 or 50 µM	Reduced dorsal and ventral tissue and no torsion at lower concentrations. Post trochal protrusion with no evident ventral, dorsal tissue o posterior.
REF[3]	Gastropoda (Mollusca)	*Tritia obsoleta*	Unequal	3D + the micromeres known to require a signal from 3D	Yes	10 µM	Post trochal protrusion with no evident ventral, dorsal tissue o posterior. Lack of eyes and velar lobes.
REF[4]	Gastropoda (Mollusca)	*Tectura scutum*	Equal	3D	Yes	10 or 50 µM	Loss or reduction of larval retractor muscles (derived from 4d) at lower concentration. No shell or operculum at higher concentrations.
REF[5]	Gastropoda (Mollusca)	*Crepidula fornicata*	Equal	Initially in the progeny of the first quartet micromeres, just prior to the birth of the third quartet (e.g., late during the 16-cell and subsequently during the 20-cell stages). Afterwards,in 3D just prior to the 24-cell stage, transiently in 4d and finally in a subset of animal micromeres immediately following those stages.	Yes	10 or 25 µM	Gastrulation defects. 1st, 2nd and 3rd quartet micromeres form protrusion with no evident ventral or dorsal tissue. No eyes or shell.
REF[6]	Gastropoda (Mollusca)	*Patella vulgata*	Equal	3D	No	10 or 50 µM	Changes in the expression of brachyury, but ventral and dorsal tissues are evident
REF[7]	Gastropoda (Mollusca)	*Testudinalia testudinalis*	Equal	3D	Unclear	10 or 40 µM	
REF[4]	Gastropoda (Mollusca)	*Lymnaea palustris*	Equal	3D	Not studied		
REF[4]	Polyplacophora (Mollusca)	*Chaetopleura apiculata*	Equal	3D	Not studied		
REF[8]	Errantia (Annelida)	*Alitta virens*	Unequal	8-cell stage at 1c, 1d and 1D; all micromeres at 16-cell (no vegetal activity); 3d and 3D activity, off in micromeres; then 1q1 and 2d descendant, 3c, 3d, 3D. Fades after that.	No	40 µM	Presumably, disrupts mesoderm bands migration to an antero-ventral position
REF[9]	Errantia (Annelida)	*Platynereis dumerilii*	Unequal	Late cleavage in nephroblasts, then later close to the blastopore.	No	10, 25 or 50 µM	Shorter trunk. Reduced musculature.
REF[10]	Sedentaria (Annelida)	*Capitella teleta*	Unequal	The earliest detectable ERK/MAPK activation is during epiboly (gastrulation) in cells positioned around the blastopore lip.	No	5, 10, 20 or 50 µM	Shorter and narrower posterior trunk. Reduced musculature.
REF[4]	Sedentaria (Annelida)	*Hydroides hexagonus*	Equal	4d	Not studied		
REF[11]	Chaetopteriformia (Annelida)	*Chaetopterus pergamentaceus*	Unequal	Not assessed	No	20 µM	Absence of hindgut, reduced musculature.
This study	Palaeoannelida (Annelida)	*Owenia fusiformis*	Equal	First in four of the most animal micromeres ($1q^{111}$) at the 3q stage (4hpf), later in 4d at the coeloblastula stage (5hpf), and at 6hpf, in six additional cells $2a^1$-$2c^1$ and $2a^2$-$2c^2$	Yes	10 µM	Antero-ventral radialisation

Supplementary Table 2. di-P-ERK1/2 activation in the coeloblastula under different treatments.

Replicate	Treatment	di-P-ERK1/2 enrichment at the coeloblastula stage (5 hpf)		n
		yes	no	
1	BFA	1	35	36
	DMSO	45	9	54
	U0126	7	47	54
	DMSO	51	12	63
2	BFA	0	120	120
	DMSO	126	1	127
	U0126	2	135	137
	DMSO	212	3	215
3	BFA	2	12	14
	DMSO	12	1	13
	U0126	4	11	15
	DMSO	27	0	27
1	SU5402	0	5	5
	DMSO	5	0	5
2	SU5402	2	45	47
	DMSO	37	5	42

Supplementary Table 3. Morphological markers and gene markers used for the characterisation of all phenotypes.

**Phenotype**	**Morphological markers**					
	Chaetae	Foregut	Hindgut	Muscles	Apical organ (AO)	Neurons in AO
**Stubby**	no	yes	yes	dorsal levators only	reduced	yes, but reduced
**Compressed**	yes	yes	yes	yes	reduced	yes, but reduced
**Radial**	no	yes	no	no	reduced	yes, but reduced
**Wild type**	yes	yes	yes	yes	yes	yes
	**Expression of gene markers in the larva**					
	six3/6	*gsc*	*cdx*	*GATA4/5/6b*	*twist*	*syt1*
**Stubby**	x	oral	hindgut	x	x	Apical neurons
**Compressed**	Oesophagus only	oral	hindgut	midgut	Trunk mesoderm	Apical neurons
**Radial**	Oesophagus only	Radially expanded	absent	midgut	Absent*	Apical neurons
**Wild type**	Apical organ and oesophagus	oral	hindgut	midgut	Trunk mesoderm	Apical neurons

* Some windows of treatment show reduced number of *twist*+ cells

Supplementary Table 4. Larval phenotypes at different concentrations of BFA and U0126 treatments.

Replicate	Treatment	Concentration (µM)	Radial n	Radial %	Wild type n	Wild type %	Total
1	BFA	0.1	3	6	47	94	50
		1	22	91,67	2	8,33	24
		5	21	95,45	1	4,55	22
		10	26	100	0	0	26
	U0126	0.1	2	8	23	92	25
		1	15	57,69	11	42,31	26
		5	25	92,59	2	7,41	27
		10	34	100	0	0	34
2	BFA	0.1	0	0	74	100	74
		1	68	100	0	0	68
		5	12	100	0	0	12
		10	35	100	0	0	35
	U0126	0.1	29	44,62	36	55,38	65
		1	63	100	0	0	63
		5	65	100	0	0	65
		10	70	100	0	0	70
	DMSO	0.1	0	0	69	100	69
		1	0	0	80	100	80
		5	0	0	74	100	74
		10	0	0	62	100	62
		10	0	0	34	100	34

Supplementary Table 5. Larval phenotypes during staggered time windows of BFA and U0126 treatments.

Treatment	Time window	Wild type		Compressed		Radial		Total
		n	%	n	%	n	%	
U0126	0.5hpf to 2c	30	88,24	0	0	4	11,76	34
	0.5hpf to 4c	19	27,14	0	0	51	72,86	70
	0.5hpf to 8c	1	1,45	0	0	68	98,55	69
	0.5hpf to 3hpf	0	0	0	0	84	100	84
	0.5hpf to 4hpf	0	0	0	0	79	100	79
	0.5hpf to 5hpf	0	0	0	0	1873	100	1873
	3hpf to 5hpf	0	0	0	0	24	100	24
	4hpf to 6hpf	0	0	0	0	517	100	517
	4hpf to 7hpf	5	23,81	0	0	16	76,19	21
BFA	0.5hpf to 2c	29	100	0	0	0	0	29
	0.5hpf to 4c	60	100	0	0	0	0	60
	0.5hpf to 8c	65	100	0	0	0	0	65
	0.5hpf to 3hpf	39	43,82	50	56,18	0	0	89
	0.5hpf to 4hpf	30	41,1	12	16,44	31	42,47	73
	0.5hpf to 5hpf	0	0	0	0	795	100	795
	3hpf to 5hpf	0	0	0	0	21	100	21
	4hpf to 6hpf	0	0	0	0	10	100	10
	4hpf to 7hpf	0	-	0	-	0	-	15

Supplementary Table 6. List of candidate genes and their gene annotation IDs in differential gene expression analyses.

Candidate	Gene Id
AP2	OFUSG17466
BAMBI	OFUSG04738
cdx	OFUSG03177
delta	OFUSG11344
fer3	OFUSG04687
foxh1	OFUSG04867
foxQ2	OFUSG09682
gsc	OFUSG11740
hand2	OFUSG03211
irxA	OFUSG05354
lhx1/5	OFUSG14965
msx2a	OFUSG05673
noggin	OFUSG18439
notch-like	OFUSG01867
POU4	OFUSG03481
POU3	OFUSG01141
rhox	OFUSG21500
six3/6	OFUSG12368
twist	OFUSG04722
wnt1	OFUSG16504
wnt4	OFUSG13755
wntA	OFUSG09779

Supplementary Table 7. Summary of scoring counts for gene expression (ISH) in coeloblastulae and larval phenotypes in control, BFA and U0126 phenotypes treated from 0.5 hpf to 5 hpf.

Treatment	Stage	Total
control	Coeloblastula	1252
	Larva	1330
U0126	Coeloblastula	1378
	Larva	1873
BFA	Coeloblastula	1594
	Larva	795

Supplementary Table 8. Genes expressed in the 4d blastomere or the 4d lineage in other spiralians.

Species	Gene/Protein	Notes	Reference
Tritia obsoleta	*cdx*	Other blastomere, weakly in 4d. Later in 4d lineage	REF[12]
	Delta	Also in MR and ML	REF[13]
	vasa	4d plus other cells, and then enriched in 4d lineage	REF[14]
	nanos	4d + 4D	REF[15]
Tubifex tubifex	*Delta*	D lineage including 4d	REF[16]
Crepidula fornicata	*vasa*	4d	REF[17]
	β-catenin	4d lineage	REF[18]

Supplementary Table 9. Gene expression (FISH) at 5.5 hpf in control, BFA and U0126 phenotypes treated from 0.5 hpf to 5 hpf.

Time window	Stage	Treatment	Gene	Wild type		Radial		No expression		Total
				n	%	n	%	n	%	
0.5hpf-5hpf	CB*	control	*gsc*	223	85,44	0	0	38	14,56	261
		U0126		11	16,42	11	16,42	15	22,39	67
		BFA		2	2,13	0	0	92	97,87	94
		control	*AP2*	27	79,41	0	0	7	20,59	34
		U0126		84	10	0	0	78	92,86	84

*Coeloblastula

Supplementary Table 10. Larval phenotypes during staggered time windows of LY411575 treatments.

Time window	Treatment	Wild type		Radial		Compressed		Stubby		Total
		n	%	n	%	n	%	n	%	%
5 hpf to 24 hpf	control	74	100	0	0	0	0	0	0	74
	LY411575	0	0	0	0	0	0	112	100	112
0.5 hpf to 5 hpf	control	45	100	0	0	0	0	0	0	45
	LY411575	68	100	0	0	0	0	0	0	68

Supplementary Table 11. Gene expression (ISH) in larval phenotypes in control and LY411575 treatments.

Stage	Treatment	Time window	Gene	Wild type n	%	Stubby n	%	Total
24 hpf	control	0.5 hpf to 5 hpf	*gsc*	23	100	0	0	23
	LY411575			37	100	0	0	37
	control	5 hpf to 24 hpf		18	100	0	0	18
	LY411575			0	0	10	100	10
	control	0.5 hpf to 5 hpf	*cdx*	3	100	0	0	3
	LY411575			18	100	0	0	18
	control	5 hpf to 24 hpf		15	100	0	0	15
	LY411575			0	0	27	100	27
	control	0.5 hpf to 5 hpf	*syt1*	20	100	0	0	20
	LY411575			13	100	0	0	13
	control	5 hpf to 24 hpf		16	100	0	0	16
	LY411575			0	0	12	100	12

Supplementary Table 12. Larval phenotypes during staggered time windows of SU5402 treatments.

Treatment	Time window	Wild type n	%	Radial n	%	Compressed n	%	Total
control	0.5 hpf to 7hpf	13	100	0	0	0	0	13
SU5402	0.5 hpf to 24hpf	0	0	0	0	23	100	23
control		49	100	0	0	0	0	49
control	3hpf-5hpf	26	100	0	0	0	0	26
SU5402		1	3,2	0	0	30	96,7	31
SU5402	4hpf-6hpf	3	6,5	43	93,5	0	0	46
control		42	100	0	0	0	0	42
control	3hpf-7hpf	87	100	0	0	0	0	87
SU5402		0	0	55	100	0	0	55

Supplementary Table 13. Role of FGF-MAPK and Notch-Delta signalling pathways in axial specification across bilaterians outside Spiralia.

Clade	Species	FGF axial	ERK axial	Notch axial	Notes	References
Echinodermata	*Paracentrotus lividus*	?	Y	?	ERK regulating nodal role in axial polarity through the ETS protein Yan/Tel	REF[19]
	Lytechinus variegatus	?	Y	Y	Another MAPK, p38, regulating nodal role in axial polarity. Delta as a vegetal organising center.	REFs[20,21]
Hemichordata	*Ptychodera flava*	?	Y	?	ERK role in dorsal tissue formation	REF[22]
	Saccoglossus kowalevskii	N	?	Y	Notch role in posterior elongation	REF[23]
Cephalochordata		N	N	N		
Tunicata	*Ciona intestinalis*	Y	Y	N	FGF-ERK role in posterior specification of epidermis	REF[24]
Craniata	*Xenopus tropicalis*	Y	Y	Y	FGF-ERK in dorsal mesoderm, regulation of *cdx* and communication with Delta-Notch for posterior elongation	REFs[25-27]
	Danio rerio	Y	Y		FGF-ERK role in dorsal fate, and later in posterior elongation	REFs[28-30]
Arthropoda	*Parasteatoda tepidariorum*	Y	?	Y	FGF role in dorsal-ventral through cumulus migration. Notch-Delta role in caudal specification and patterning	REFs[31,32]
Nematoda	*Caenorhabditis elegans*	?	?	Y	Notch-Delta maternal role in setting up posterior and dorsal-ventral identities	REF[33]

Supplementary References

1 Vellutini, B. C., Martin-Duran, J. M. & Hejnol, A. Cleavage modification did not alter blastomere fates during bryozoan evolution. *BMC Biol* **15**, 33 (2017).

2 Koop, D., Richards, G. S., Wanninger, A., Gunter, H. M. & Degnan, B. M. The role of MAPK signaling in patterning and establishing axial symmetry in the gastropod *Haliotis asinina*. *Dev Biol* **311**, 200-212 (2007).

3 Lambert, J. D. & Nagy, L. M. MAPK signaling by the D quadrant embryonic organizer of the mollusc *Ilyanassa obsoleta*. *Development* **128**, 45-56 (2001).

4 Lambert, J. D. & Nagy, L. M. The MAPK cascade in equally cleaving spiralian embryos. *Dev Biol* **263**, 231-241 (2003).

5 Henry, J. J. & Perry, K. J. MAPK activation and the specification of the D quadrant in the gastropod mollusc, *Crepidula fornicata*. *Dev Biol* **313**, 181-195 (2008).

6 Lartillot, N., Lespinet, O., Vervoort, M. & Adoutte, A. Expression pattern of *Brachyury* in the mollusc *Patella vulgata* suggests a conserved role in the establishment of the AP axis in Bilateria. *Development* **129**, 1411-1421 (2002).

7 Kozin, V. V., Babakhanova, R. A. & Kostiuchenko, R. P. Functional role for MAP kinase signaling in cell lineage and dorso-ventral axis specification in the basal gastropod T*estudinalia testudinalis* (Patellogastropoda, Molluska). *Ontogenez* **44**, 42-56 (2013).

8 Kozin, V. V., Filimonova, D. A., Kupriashova, E. E. & Kostyuchenko, R. P. Mesoderm patterning and morphogenesis in the polychaete *Alitta virens* (Spiralia, Annelida): Expression of mesodermal markers *Twist*, *Mox*, *Evx* and functional role for MAP kinase signaling. *Mech Dev* **140**, 1-11 (2016).

9 Pfeifer, K., Schaub, C., Domsch, K., Dorresteijn, A. & Wolfstetter, G. Maternal inheritance of twist and analysis of MAPK activation in embryos of the polychaete annelid *Platynereis dumerilii*. *PLoS One* **9**, e96702 (2014).

10 Amiel, A. R., Henry, J. Q. & Seaver, E. C. An organizing activity is required for head patterning and cell fate specification in the polychaete annelid *Capitella teleta*: new insights into cell-cell signaling in Lophotrochozoa. *Dev Biol* **379**, 107-122 (2013).

11 Lanza, A. R. & Seaver, E. C. Activin/Nodal signaling mediates dorsal-ventral axis formation before third quartet formation in embryos of the annelid *Chaetopterus pergamentaceus*. *Evodevo* **11**, 17 (2020).

12 Johnson, A. B. & Lambert, J. D. The Caudal ParaHox gene is required for hindgut development in the mollusc *Tritia* (a.k.a. *Ilyanassa*). *Dev Biol* **470**, 1-9 (2021).

13 Gharbiah, M., Nakamoto, A., Johnson, A. B., Lambert, J. D. & Nagy, L. M. *Ilyanassa* Notch signaling implicated in dynamic signaling between all three germ layers. *Int J Dev Biol* **58**, 551-562 (2014).

14 Swartz, S. Z., Chan, X. Y. & Lambert, J. D. Localization of *Vasa* mRNA during early cleavage of the snail *Ilyanassa*. *Dev Genes Evol* **218**, 107-113 (2008).

15 Rabinowitz, J. S., Chan, X. Y., Kingsley, E. P., Duan, Y. & Lambert, J. D. *Nanos* is required in somatic blast cell lineages in the posterior of a mollusk embryo. *Curr Biol* **18**, 331-336 (2008).

16 Matsuo, K., Yoshida, H. & Shimizu, T. Differential expression of caudal and dorsal genes in the teloblast lineages of the oligochaete annelid *Tubifex tubifex*. *Dev Genes Evol* **215**, 238-247 (2005).

17 Henry, J. J., Perry, K. J., Fukui, L. & Alvi, N. Differential localization of mRNAs during early development in the mollusc, *Crepidula fornicata*. *Integr Comp Biol* **50**, 720-733 (2010).

18 Henry, J. Q., Perry, K. J. & Martindale, M. Q. beta-catenin and early development in the gastropod, *Crepidula fornicata*. *Integr Comp Biol* **50**, 707-719 (2010).

19 Molina, M. D. *et al.* MAPK and GSK3/ss-TRCP-mediated degradation of the maternal Ets domain transcriptional repressor Yan/Tel controls the spatial expression of nodal in the sea urchin embryo. *PLoS Genet* **14**, e1007621 (2018).

20 Sweet, H. C., Gehring, M. & Ettensohn, C. A. LvDelta is a mesoderm-inducing signal in the sea urchin embryo and can endow blastomeres with organizer-like properties. *Development* **129**, 1945-1955 (2002).

21 Bradham, C. A. & McClay, D. R. p38 MAPK is essential for secondary axis specification and patterning in sea urchin embryos. *Development* **133**, 21-32 (2006).

22 Rottinger, E., DuBuc, T. Q., Amiel, A. R. & Martindale, M. Q. Nodal signaling is required for mesodermal and ventral but not for dorsal fates in the indirect developing hemichordate, *Ptychodera flava*. *Biol Open* **4**, 830-842 (2015).

23 Fritzenwanker, J. H., Uhlinger, K. R., Gerhart, J., Silva, E. & Lowe, C. J. Untangling posterior growth and segmentation by analyzing mechanisms of axis elongation in hemichordates. *Proc Natl Acad Sci U S A* **116**, 8403-8408 (2019).

24 Pasini, A., Manenti, R., Rothbacher, U. & Lemaire, P. Antagonizing retinoic acid and FGF/MAPK pathways control posterior body patterning in the invertebrate chordate *Ciona intestinalis*. *PLoS One* **7**, e46193 (2012).

25 Curran, K. L. & Grainger, R. M. Expression of activated MAP kinase in *Xenopus laevis* embryos: evaluating the roles of FGF and other signaling pathways in early induction and patterning. *Dev Biol* **228**, 41-56 (2000).

26 Keenan, I. D., Sharrard, R. M. & Isaacs, H. V. FGF signal transduction and the regulation of Cdx gene expression. *Dev Biol* **299**, 478-488 (2006).

27 Hubaud, A. & Pourquie, O. Signalling dynamics in vertebrate segmentation. *Nat Rev Mol Cell Biol* **15**, 709-721 (2014).

28 Fürthauer, M., Thisse, C. & Thisse, B. A role for FGF-8 in the dorsoventral patterning of the zebrafish gastrula. *Development* **124**, 4253-4264 (1997).

29 Furthauer, M., Van Celst, J., Thisse, C. & Thisse, B. Fgf signalling controls the dorsoventral patterning of the zebrafish embryo. *Development* **131**, 2853-2864 (2004).

30 Stulberg, M. J., Lin, A., Zhao, H. & Holley, S. A. Crosstalk between Fgf and Wnt signaling in the zebrafish tailbud. *Dev Biol* **369**, 298-307 (2012).

31 Oda, H. *et al.* Progressive activation of Delta-Notch signaling from around the blastopore is required to set up a functional caudal lobe in the spider *Achaearanea tepidariorum*. *Development* **134**, 2195-2205 (2007).

32 Wang, R., Karadas, L., Schiffer, P. & Pechmann, M. FGF signalling is involved in cumulus migration in the common house spider *Parasteatoda tepidariorum*. *bioRxiv*, 2021.2010.2001.462731 (2021).

33 Mello, C. C., Draper, B. W. & Priess, J. R. The maternal genes *apx-1* and *glp-1* and establishment of dorsal-ventral polarity in the early *C. elegans* embryo. *Cell* **77**, 95-106 (1994).
