# Supplementary material for: ERK1/2 is an ancestral organising signal in spiral cleavage
Source: Nat Commun. 2022 Apr 28;13:2286. doi: 10.1038/s41467-022-30004-4 (PMC9050690; doi:10.1038/s41467-022-30004-4)
Supplement: Supplementary file 3 — Description of Additional Supplementary Files [file 41467_2022_30004_MOESM3_ESM.pdf]

Description of Additional Supplementary Files

File Name: Supplementary Data 1
Description: Differential Expression Analyses, KEGG enrichment, phenotype assessment and multiple protein alignments.

File Name: Supplementary Data 2
Description: Gene Ontology term enrichment analyses.
