# Supplementary material for: ERK1/2 is an ancestral organising signal in spiral cleavage
Source: Nat Commun. 2022 Apr 28;13:2286. doi: 10.1038/s41467-022-30004-4 (PMC9050690; doi:10.1038/s41467-022-30004-4)
Supplement: Supplementary file 6 — Reporting Summary [file 41467_2022_30004_MOESM6_ESM.pdf]

nature portfolio

Corresponding author(s): J.M. Martin-Duran

Last updated by author(s): Apr 2, 2022

Reporting Summary

Nature Portfolio wishes to improve the reproducibility of the work that we publish. This form provides structure for consistency and transparency in reporting. For further information on Nature Portfolio policies, see our Editorial Policies and the Editorial Policy Checklist.

Statistics

For all statistical analyses, confirm that the following items are present in the figure legend, table legend, main text, or Methods section.

n/a	Confirmed	
☐	☒	The exact sample size (*n*) for each experimental group/condition, given as a discrete number and unit of measurement
☐	☒	A statement on whether measurements were taken from distinct samples or whether the same sample was measured repeatedly
☐	☒	The statistical test(s) used AND whether they are one- or two-sided<br>*Only common tests should be described solely by name; describe more complex techniques in the Methods section.*
☐	☒	A description of all covariates tested
☐	☒	A description of any assumptions or corrections, such as tests of normality and adjustment for multiple comparisons
☐	☒	A full description of the statistical parameters including central tendency (e.g. means) or other basic estimates (e.g. regression coefficient) AND variation (e.g. standard deviation) or associated estimates of uncertainty (e.g. confidence intervals)
☐	☒	For null hypothesis testing, the test statistic (e.g. *F*, *t*, *r*) with confidence intervals, effect sizes, degrees of freedom and *P* value noted<br>*Give P values as exact values whenever suitable.*
☒	☐	For Bayesian analysis, information on the choice of priors and Markov chain Monte Carlo settings
☒	☐	For hierarchical and complex designs, identification of the appropriate level for tests and full reporting of outcomes
☒	☐	Estimates of effect sizes (e.g. Cohen's *d*, Pearson's *r*), indicating how they were calculated

Our web collection on statistics for biologists contains articles on many of the points above.

Software and code

Policy information about availability of computer code

Data collection	Images were collected using: Infinite Analyze v.7.02.920, LAS AF. Z projections were generated using: Fiji ImageJ 1.53c.
Data analysis	The following publicly available code was used in this study: pheatmap v.1.0.12, HOMER v.4.11, TOBIAS v.0.12.0, pyGenomeTracks v.2.1, Kallisto v.0.44.0, DEseq2 v.1.38.0, GOseq v.1.40.0, MAFFT v.7, gBlocks v.0.91b, RAxML v.8.2.11, FastTree 2.1.11, InterProScan 5, FigTree v.1.4.4

For manuscripts utilizing custom algorithms or software that are central to the research but not yet described in published literature, software must be made available to editors and reviewers. We strongly encourage code deposition in a community repository (e.g. GitHub). See the Nature Portfolio guidelines for submitting code & software for further information.

Data

Policy information about availability of data

All manuscripts must include a data availability statement. This statement should provide the following information, where applicable:

- Accession codes, unique identifiers, or web links for publicly available datasets
- A description of any restrictions on data availability
- For clinical datasets or third party data, please ensure that the statement adheres to our policy

The authors declare that the data supporting the findings of this study are available within the paper and its supplementary information files. All raw RNA-seq sequencing data generated in this study is available in the European Nucleotide Archive (ENA) under accession number PRJEB47195 (https://www.ebi.ac.uk/ena/browser/view/PRJEB47195). Additionally, this study used the genome assembly and annotation of Owenia fusiformis, available at ENA under accession GCA_903813345 and ATAC-seq peaks available at Gene Expression Omnibus with accession number GSE184126 and a public repository (https://github.com/ChemaMD/OweniaGenome).

Field-specific reporting

Please select the one below that is the best fit for your research. If you are not sure, read the appropriate sections before making your selection.

- [x] Life sciences
- [ ] Behavioural & social sciences
- [ ] Ecological, evolutionary & environmental sciences

For a reference copy of the document with all sections, see nature.com/documents/nr-reporting-summary-flat.pdf

Life sciences study design

All studies must disclose on these points even when the disclosure is negative.

Sample size	For RNA-seq analyses, sample sizes were estimated based on the amount of total RNA obtained for subsequent library prep (at least 100 ng). In gene expression analyses, immunohistochemistry and drug inhibition experiments, sample sizes were calculated to ensure consistency of expression/localisation patterns and phenotypic outcomes (number of embryos assessed are always provided throughout the manuscript and in supplementary data).
Data exclusions	No data was excluded from the study.
Replication	All gene expression analyses, protein immunolocalisation and drug inhibitions were performed at least in duplicate. Four replicates were collected for RNA-seq differential expression analyses. All experiments were reproducible.
Randomization	All embryonic samples used in this study were collected and assigned to experimental conditions randomly.
Blinding	Given that the microscopic size of the embryos of O. fusiformis does not allow to identify phenotypes a priori, blind collection was not required in this study.

Reporting for specific materials, systems and methods

We require information from authors about some types of materials, experimental systems and methods used in many studies. Here, indicate whether each material, system or method listed is relevant to your study. If you are not sure if a list item applies to your research, read the appropriate section before selecting a response.

Materials & experimental systems

n/a	Involved in the study	
[ ]	[x]	Antibodies
[x]	[ ]	Eukaryotic cell lines
[x]	[ ]	Palaeontology and archaeology
[ ]	[x]	Animals and other organisms
[x]	[ ]	Human research participants
[x]	[ ]	Clinical data
[x]	[ ]	Dual use research of concern

Methods

n/a	Involved in the study	
[x]	[ ]	ChIP-seq
[x]	[ ]	Flow cytometry
[x]	[ ]	MRI-based neuroimaging

Antibodies

Antibodies used	anti-di-P-ERK1/2 (Sigma-Aldrich, #M8159), anti-acetylated α-tubulin (clone 6-11B-1, Millipore-446 Sigma, #MABT868), anti-mouse peroxidase (POD) (Millipore-Sigma, #111207733910), anti-mouse AlexaFluor 594 (ThermoFisher Scientific, #A32731)
Validation	These antibodies are routinely used in the study of spiral cleaving embryos (references provided in the manuscript). Validation of the anti-di-P ERK1/2 was done using a negative control.

Animals and other organisms

Policy information about studies involving animals; ARRIVE guidelines recommended for reporting animal research

Laboratory animals	The study did not involve laboratory animals
Wild animals	The study did not involve wild animals

Field-collected samples	The study involved embryonic samples obtained in the lab through in vitro fertilisation of mature adults of the annelid species Owenia fusiformis, collected from the coast near the Station Biologique de Roscoff (France) during the reproductive season (May to July) at low tide. In the lab, animals were kept in artificial seawater (ASW) at 15°C with a 16:8 (light:darkness) photperiod. In vitro fertilizations were conducted as previously described (references provided) and embryos develop in glass bowls with filtered ASW at 19 oC until the desired embryonic stage. Adult worms were anesthetized with magnesium chloride and sacrificed in ethanol at the end of experiments.
Ethics oversight	No ethical approval was required to work with annelid worms.

Note that full information on the approval of the study protocol must also be provided in the manuscript.
